# VDAC1 Negatively Regulates Floral Transition in *Arabidopsis thaliana*

**DOI:** 10.3390/ijms222111603

**Published:** 2021-10-27

**Authors:** Jingya Xu, Yuzhen Zhang, Hongjia Ren, Runyi Yu, Chen Yuan, Yikai Hu, Rumeng Xu, Xuming Wang, Cheng Qin

**Affiliations:** 1 College of Life and Environmental Sciences, Hangzhou Normal University, Hangzhou 311121, China; xujingya@stu.hznu.edu.cn (J.X.); yuzhenzhang1010@gmail.com (Y.Z.); reonia1733@gmail.com (H.R.); yurunyi99@gmail.com (R.Y.); rachelfebruary68@gmail.com (C.Y.); h385160811@gmail.com (Y.H.); 2 State Key Laboratory for Managing Biotic and Chemical Threats to the Quality and Safety of Agro-Products, Zhejiang Academy of Agricultural Sciences, Hangzhou 310021, China; rmengxu@gmail.com (R.X.); xmwang@zaas.ac.cn (X.W.)

**Keywords:** VDAC1, flowering locus T, floral transition, early flowering, *Arabidopsis*

## Abstract

Voltage-dependent anion channels (VDACs) are the most important proteins in mitochondria. They localize to the outer mitochondrial membrane and contribute to the metabolite transport between the mitochondria and cytoplasm, which aids plant growth regulation. Here, we report that *Arabidopsis thaliana* VDAC1 is involved in the floral transition, with the loss of *AtVDAC1* function, resulting in an early-flowering phenotype. *AtVDAC1* is expressed ubiquitously in *Arabidopsis*. To identify the flowering pathway integrators that may be responsible for AtVDAC1′s function during the floral transition, an RNA-seq analysis was performed. In total, 106 differentially expressed genes (DEGs) were identified between wild-type and *atvdac1-5* mutant seedlings. However, none were involved in flowering-related pathways. In contrast, AtVDAC1 physically associated with FLOWERING LOCUS T. Thus, in the floral transition, AtVDAC1 may function partly through the FLOWERING LOCUS T protein.

## 1. Introduction

Voltage-dependent anion channels (VDACs) are the most abundant proteins in the mitochondria, especially in the outer mitochondrial membrane, in which 34.2% of the surface consists of VDAC proteins [1]. VDAC proteins are crucial for both mitochondrial functions and cellular energy transactions because they play important roles in metabolite transport between the mitochondria and cytoplasm [2,3]. Several studies have identified VDAC isoforms in plants, such as wheat [4,5], rice [6,7,8], *Lotus japonicas* [9], tobacco [10], and *Arabidopsis thaliana* [11,12,13].

There are five VDAC isoforms in *Arabidopsis*, four of which have been characterized [12,13]. AtVDAC1 is involved in reproductive development. In particular, the *atvdac1* knockout mutants exhibit slender and shorter siliques, resulting in a reduced seed set [13,14]. In *atvdac1* plants, pollen development, including the number of pollen grains, pollen germination rate, and tube length of germinated pollen, is affected [13,15]. This suggests that zygotic or early embryo lethality may be the main reason for the decreased seed production in the *atvdac1* mutant [12]. The *atvdac1* mutant also has a deficiency in female development, resulting in undeveloped ovules in siliques. Furthermore, the mitochondrial transmembrane potential and ATP synthesis rate are impacted [2]. In *Arabidopsis*, VDAC1 negatively regulates cold responses, with the *atvdac1* mutant showing a more rapid germination rate and enhanced tolerance under cold-stress conditions [16]. AtVDAC1 is also involved in *Agrobacterium tumefaciens* infection tolerance, given that AtVDAC1-overexpression seedlings demonstrate enhanced tolerance to *Agrobacterium* infections by suppressing apoptotic stress [17].

In *Arabidopsis*, *VDAC2* and *VDAC4* play important roles in plant growth, leaf development, reactive oxygen species (ROS) accumulation, and mitochondrial transmembrane potential [12,13,14]. For instance, the VDAC2 function is required for plant disease defenses [18,19], and the *atvdac2* mutation will result in abscisic acid insensitivity [20].

Although *atvdac3* mutants are not distinguishable from wild type (WT) under standard conditions, AtVDAC3 interacts with several proteins in *Arabidopsis* [21,22,23]. It specifically interacts with kinesin-like protein 1 to stabilize ATP levels and balance the aerobic respiration pathways during seed germination at low temperatures [21]. AtVDAC3 also interacts with thioredoxin m2 (AtTrx m2) and Metallothionein 2b (AtMT2b), which are both involved in ROS production under salt stress. Overexpression of *AtVDAC3* results in short primary roots and increased ROS accumulation levels under NaCl stress conditions, with AtTrx m2 and AtMT2b having opposite roles [22,23]. Furthermore, VDAC3 modifies the size and number of mitochondria and regulates root growth in phosphorus- and Fe-deficient conditions [24,25].

According to this research, VDACs appear to be vital in plant growth. However, the specific functions of each isoform, particularly those of VDACs implicated in flowering time, are still unknown. Here, we demonstrated that AtVDAC1 functioned as a repressor during floral transition, and the loss of AtVDAC1 function resulted in an early-flowering phenotype. However, none of the expression levels of key flowering pathway integrators were changed in *atvdac1-5* mutant seedlings. In contrast, AtVDAC1 physically associated with FLOWERING LOCUS T (FT). Collectively, our results suggest that AtVDAC1 functions in the floral transition partly through the FT protein.

## 2. Results

### 2.1. AtVDAC1 Participates in Flowering-Time Regulation in Arabidopsis

**Figure 1:**
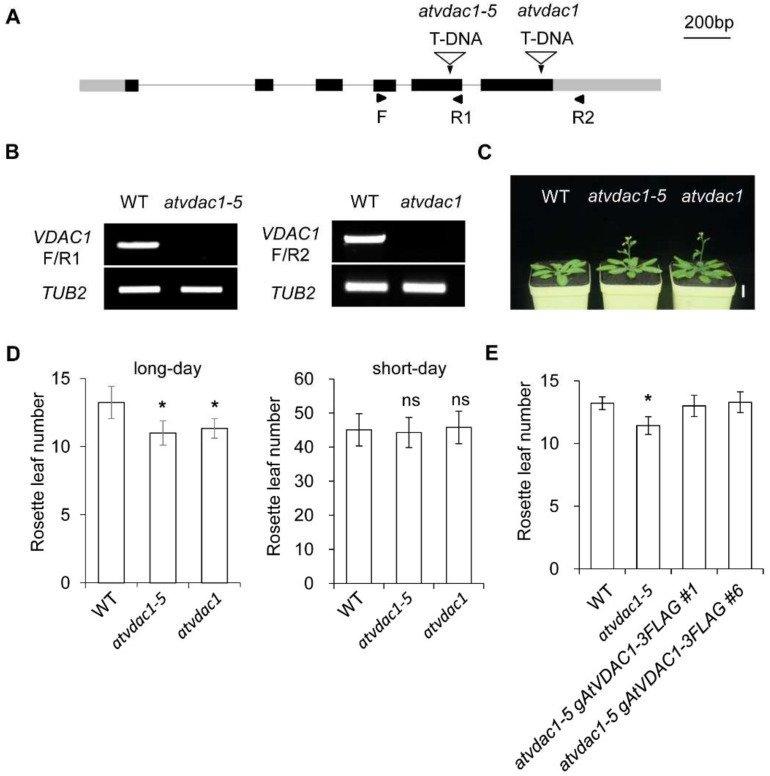
*VDAC1* represses flowering time in *Arabidopsis thaliana* (**A**) Schematic diagram showing the *AtVDAC1* genomic structure and the T-DNA insertion site in *atvdac1-5* (SALK_058473) and *atvdac1* (SALK_034653). Exons are represented by black boxes, gray boxes represent untranslated regions, and black lines indicate introns. Arrowheads indicate the positions of primers used in (**B**). (**B**) *AtVDAC1* transcripts were undetectable in *atvdac1-5* and *atvdac1* by semi-quantitative RT-PCR using the primers shown in (**A**). *TUB2* was amplified as an internal control. (**C**) *atvdac1-5* and *atvdac1* show early-flowering phenotypes compared with wild-type plants under long-day conditions. Scale bar: 2 cm. (**D**) Flowering time of *atvdac1-5* and *atvdac1* grown under long-day and short-day conditions. Values were scored from at least 15 plants per genotype. Error bars indicate standard deviations (SDs). Asterisks indicate significant differences (Student’s *t* test, *p* < 0.05). (**E**) Flowering time of two independent transgenic lines carrying the *gAtVDAC1-3FLAG* construct in the *atvdac1-5* background grown under long-day conditions. Values were scored from at least 15 plants of each genotype. Error bars indicate standard deviations (SDs). Asterisks indicate significant differences (Student’s *t* test, *p* < 0.05).

VDACs play important roles in plant growth, but whether VDACs are involved in flowering-time control is unknown. To identify the functions of *AtVDAC1* during the floral transition, we used the *atvdac1-5* mutant (SALK_058473) and *atvdac1* mutant (SALK_034653), which contain an insertion in the fifth exon and sixth exon, respectively (Figure 1A). They were identified as knock-out mutants (Figure 1B). *atvdac1-5* and *atvdac1* exhibited early-flowering phenotypes compared to WT seedlings under long-day conditions (Figure 1C,D), while *atvdac1-5* and *atvdac1* displayed comparable flowering times to WT seedlings under short-day conditions (Figure 1D), suggesting that *AtVDAC1* inhibits the floral transition.

To verify that the loss of AtVDAC1 function is responsible for the early-flowering phenotype of *atvdac1-5*, we transformed the *atvdac1-5* line with the *gAtVDAC1-3FLAG* construct. Two independent *atvdac1-5 gAtVDAC1-3FLAG* transformants exhibited comparable flowering times to WT plants (Figure 1E). These observations suggest that AtVDAC1 acts as a flowering repressor.

### 2.2. AtVDAC1 Is Expressed Ubiquitously in Arabidopsis

**Figure 2:**
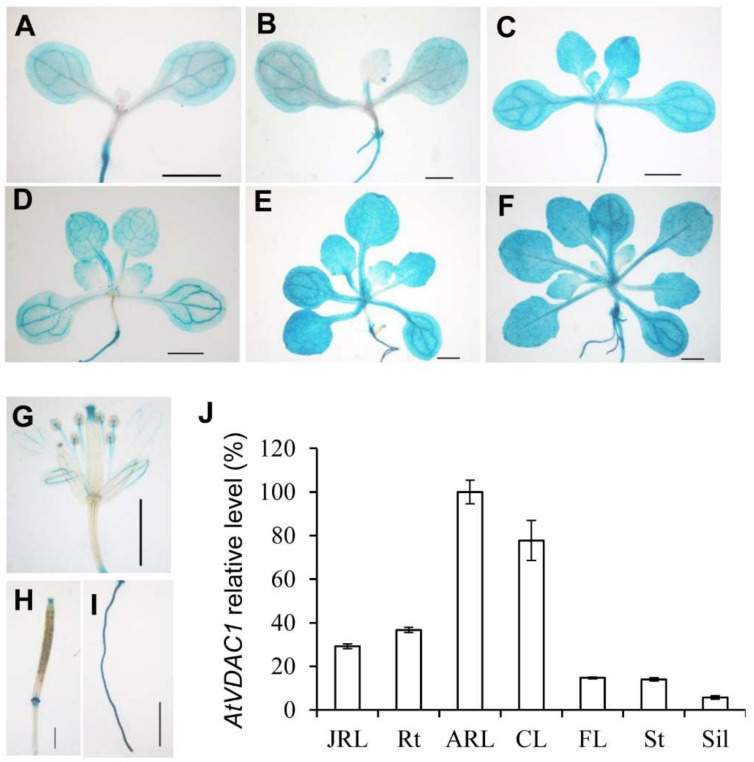
*AtVDAC1* is Expressed Ubiquitously in *Arabidopsis thaliana* (**A**–**H**) Representative GUS staining of *gAtVDAC1-GUS* transgenic plants displaying *AtVDAC1* expression in seedlings at 3 (**A**), 5 (**B**), 7 (**C**), 9 (**D**), 11 (**E**), and 13 (**F**) DAG, as well as in a flower (**G**), silique (**H**), and root (**I**). Scale bars: 1 mm. (**J**) Expression analysis of *AtVDAC1* in various tissues of WT seedlings. Expression levels are shown as relative values to the maximum level, set at 100%. JRL, juvenile rosette leaves; Rt, roots; ARL, adult rosette leaves; CL, cauline leaves; FL, flowers; St, inflorescence stems; Sil, siliques.

To examine the expression patterns of *AtVDAC1* in *Arabidopsis*, a *gAtVDAC1-GUS* reporter construct was generated. Almost all of the *gAtVDAC1-GUS* transgenic lines showed similar GUS staining patterns. We then used one representative *gAtVDAC1-GUS* transgenic line for further investigations. This line showed a strong staining pattern in leaves of developing seedlings before and during the floral transition, which occurred 3 to 13 days after germination (DAG) under long-day conditions (Figure 2A–F). The GUS signal was also detected in open flowers (Figure 2G), silique (Figure 2H), and roots (Figure 2I). This GUS staining pattern was consistent with the quantitative real-time PCR analysis, in which the highest *VDAC1* expression level occurred in leaves (Figure 2J). Thus, AtVDAC1 is active in the leaves of developing seedlings.

### 2.3. Transcriptome Profiles and Differentially Expressed Gene Identification between WT and atvdac1-5 Mutant Seedlings

To identify the downstream genes of *AtVDAC1* that might be responsible for its role in repressing flowering, RNA-seq analyses were performed between the WT and *atvdac1-5* mutant seedlings at 9 DAG. Six RNA-seq libraries from the WT and *atvdac1-5* mutant seedlings were constructed for transcriptome sequencing. The raw data were qualified, and adapters were removed, yielding approximately 6.68 Gb of sequence data from each library (Table S1). Pair-wise, Pearson’s correlation coefficients of the three biological replicates of each sample indicated that the sequencing data were highly repeatable (Figure S1).

**Figure 3:**
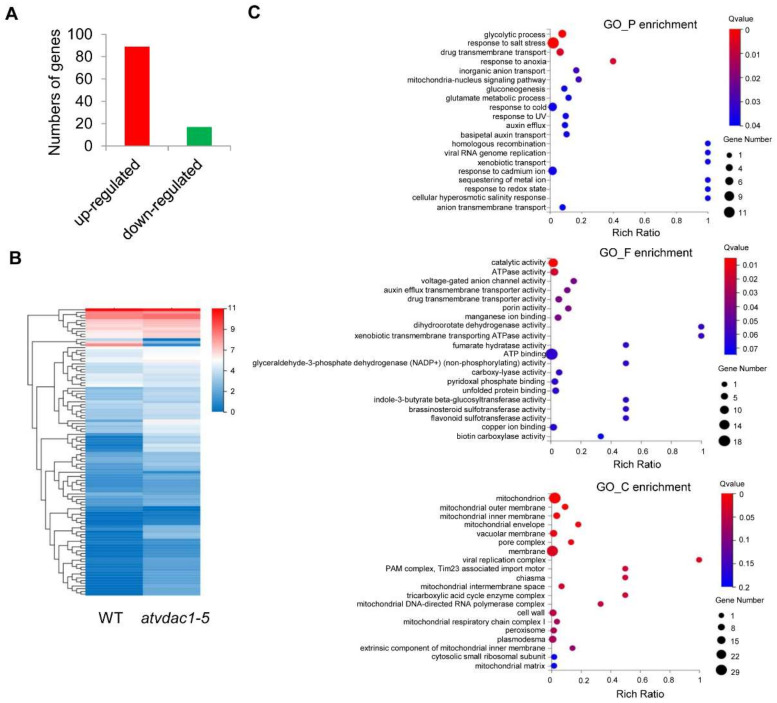
Transcriptional profiles in WT and *atvdac1-5* mutant seedlings at 9 DAG (**A**) The numbers of genes that were up-regulated or down-regulated between WT and *atvdac1-5* mutant seedlings at 9 DAG. (**B**) Expression profiles of the DEGs between WT and *atvdac1-5* mutant seedlings at 9 DAG shown using a heatmap. (**C**) GO term enrichment analysis of the DEGs between WT and *atvdac1-5* mutant seedlings.

The differentially expressed genes (DEGs) between WT and *atvdac1-5* mutant seedlings at 9 DAG were assessed using calculated reads per kilobase per million (RPKM) values. As a result, 106 DEGs were identified, with 89 and 17 genes induced and repressed, respectively (Figure 3A). The expression profiles of the 106 DEGs are shown using a heatmap (Figure 3B). To understand the functions of the DEGs, a gene ontology (GO) term enrichment analysis was performed. The dominant GO terms in biological process were “glycolytic process”, “response to salt stress”, “drug transmembrane transport”, “response to anoxia”, and “inorganic anion transport”; in molecular function, the five dominant GO terms were “catalytic activity”, “ATPase activity”, “voltage-gated anion channel activity”, “auxin efflux transmembrane transporter activity”, and “drug transmembrane transporter activity”; and in cellular component, the dominant GO terms were “mitochondrion”, “mitochondrial outer membrane”, “mitochondrial inner membrane”, “mitochondrial envelope”, and “vacuolar membrane” (Figure 3C).

We then attempted to identify the flowering-related DEGs between WT and *atvdac1-5* mutant seedlings. Surprisingly, among the 106 DEGs that were identified (Figure 3), none were involved in flowering time-related pathways.

**Figure 4:**
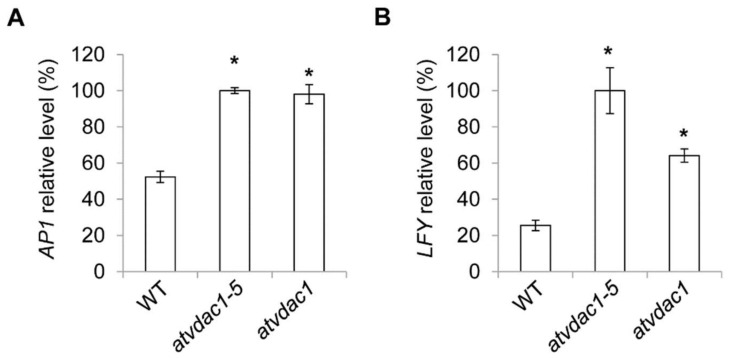
(**A**,**B**) Expression levels of *AP1* (**A**) and *LFY* (**B**) in developing wild-type (WT), *atvdac1-5* and *atvdac1* seedlings grown under long-day conditions at 13 DAG. Expression levels are shown as relative values to the maximum level, set at 100%. Error bars indicate standard deviations (SDs). Asterisks indicate significant differences (Student’s *t* test, *p* < 0.05).

Although the expression levels of the floral meristem identity genes are unaffected in *vdac1-5* mutant at 9 DAG, this could be because *AP1* and *LFY* expression levels were all low at that time. The expression levels of these genes were subsequently measured at 13 DAG, and we found that the expression of *AP1* and *LFY* were all increased in *vdac1-5* and *atvdac1* mutants (Figure 4A,B).

### 2.4. AtVDAC1 Interacts with FT

**Figure 5:**
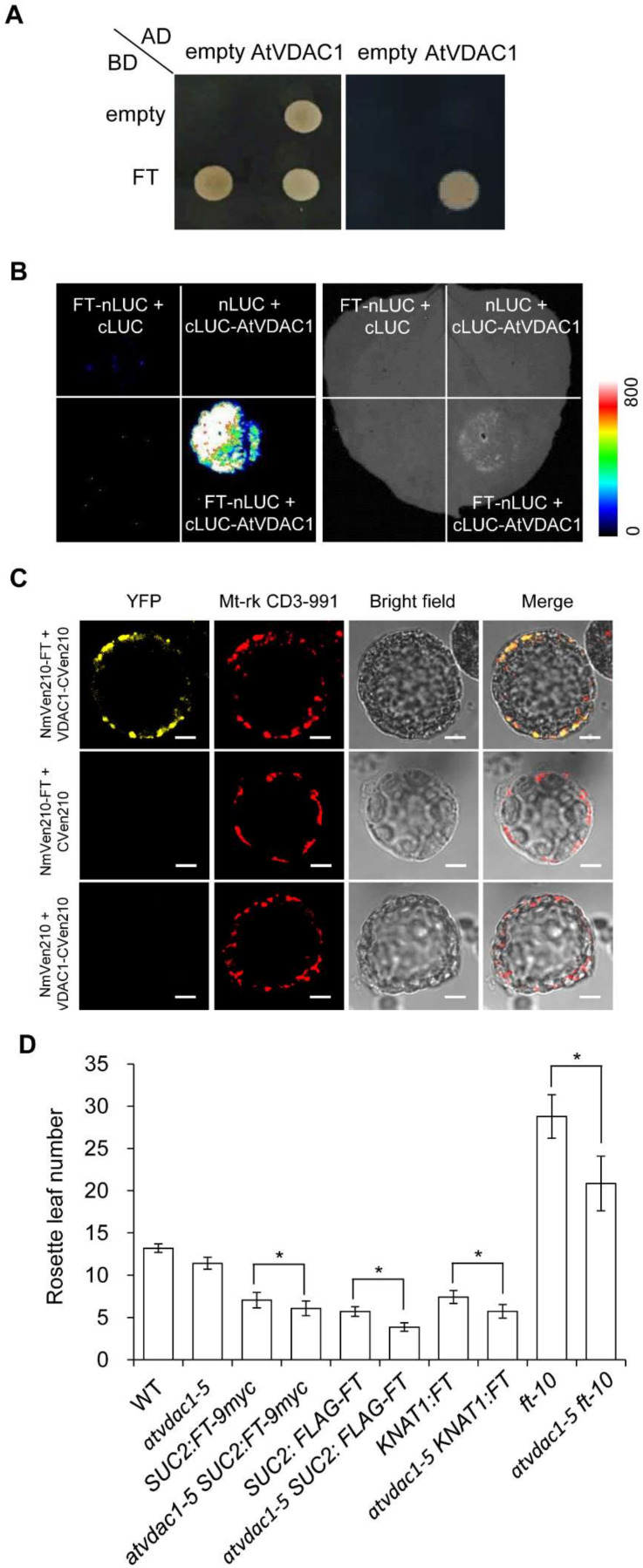
AtVDAC1 interacts with FT (**A**) Yeast two-hybrid assay confirming the interaction between AtVDAC1 and FT. Transformed yeast cells harboring FT and AtVDAC1 were grown on SD/−Leu/−Trp medium (left panel) and SD/−Ade/−His/−Leu/−Trp medium (right panel). (**B**) LCI assay showing the interaction between AtVDAC1 and FT in *Nicotiana benthamiana* leaves. The FT-nLUC and cLUC-AtVDAC1 constructs were transiently infiltrated into *N. benthamiana* leaves, and the luminescence intensity was detected using ROPER CA2048B. nLUC and cLUC were used as negative controls. The color bar shows the luminescence intensity range. (**C**) BiFC analysis of the interaction between AtVDAC1 and FT in *Arabidopsis* protoplasts. Yellow fluorescence protein (YFP), fluorescence of yellow fluorescent protein; Mt-rk CD3-991, mitochondria-mCherry marker; Merge, merge of YFP, Mt-rk CD3-991, and bright field. Scale bar: 20 μm. (**D**) Flowering times of various mutants grown under long-day conditions. Values were scored from at least 15 plants per genotype. Error bars indicate standard deviations (SDs). Asterisks indicate significant differences (Student’s *t* test, *p* < 0.05).

To elucidate how AtVDAC1 affects flowering time, we performed a yeast two-hybrid screening to identify interacting partners of AtVDAC1, and one of the interactors encoded FT. To confirm the interaction between FT and AtVDAC1, we performed several experiments. First, yeast two-hybrid assays confirmed that FT interacted with AtVDAC1 (Figure 5A). Second, FT interacted with AtVDAC1 in *Nicotiana benthamiana* leaves, as assessed by a luciferase complementation imaging (LCI) assay (Figure 5B). Third, we further performed bimolecular fluorescence complementation (BiFC) analyses and found the interaction between AtVDAC1 and FT in *Arabidopsis* protoplasts (Figure 5C). Thus, AtVDAC1 interacts with FT in plant cells.

To further investigate the relationship between AtVDAC1 and FT, a genetic analysis was performed. As a result, *atvdac1-5* enhanced the early-flowering phenotype of *SUC2:FT-9myc*, *SUC2:FLAG-FT*, and *KNAT1:FT* transgenic alleles, which indicated that VDAC1 is active in the leaves and SAM (shoot apical meristem). Furthermore, *atvdac1-5 ft-10* double mutants flowered earlier than *ft-10* (Figure 5D), indicating that AtVDAC1 also affects flowering through other independent targets.

**Figure 6:**
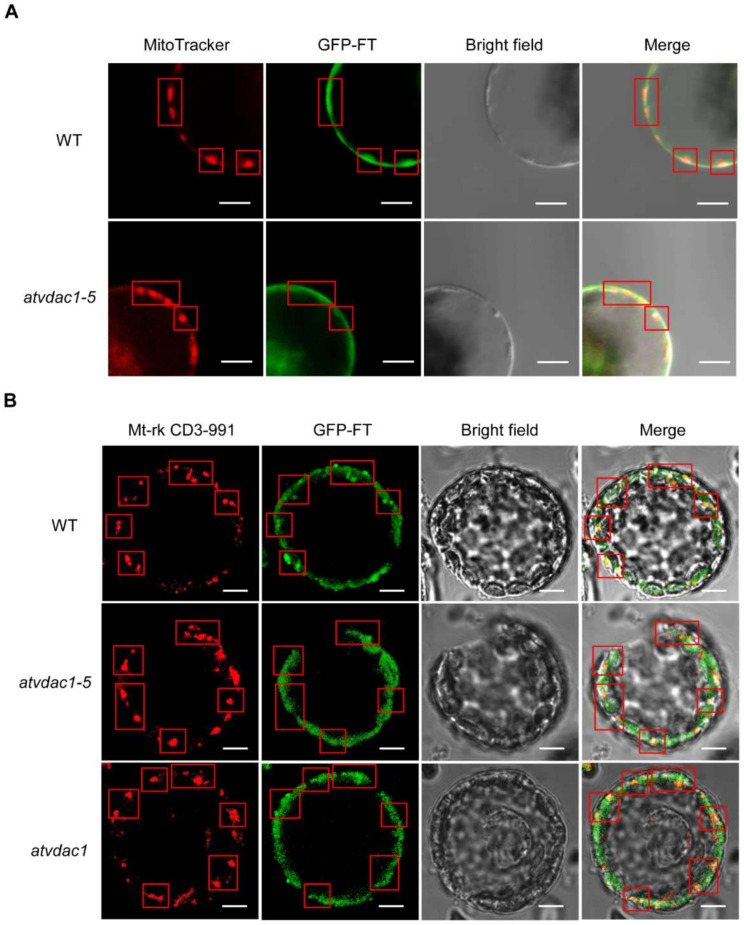
Subcellular localization of GFP-FT in WT, *atvdac1-5*, and *atvdac1* protoplast. *Arabidopsis* protoplasts were transfected with SUC2:GFP-FT. Mitochondrial marker, MitoTracker (**A**) or Mt-rk CD3-991 (**B**). Scale bar: 20 μm. Red rectangles indicated the location of mitochondria.

To further prove that FT protein movement may be the main reason for the early-flowering phenotype of *atvdac1-5* and *atvdac1*, we analyzed the FT protein localization in WT, *atvdac1-5*, and *atvdac1* mutants. As a result, we found that FT-GFP protein was visible in the mitochondria in WT, *atvdac1-5*, and *atvdac1* mutant plants (Figure 6). However, it is likely that there was a reduced level of FT-GFP signals in the mitochondria of *atvdac1–5* and *atvdac1* mutants compared to that in WT (Figure 6), indicating that AtVDAC1 may hold FT proteins in the outer membrane of mitochondria. Simultaneously, FT-GFP signals were still visible in the mitochondria in *atvdac1-5* and *atvdac1* mutants, indicating that other components such as AtVDAC2, 3, 4, homologs of AtVDAC1, may also interact with FT for holding it in mitochondria.

## 3. Discussion

The VDAC proteins play important roles in metabolite transport between the mitochondria and cytoplasm, which is crucial for both mitochondrial functions and cellular energy transactions [2,3]. They also play important roles in plant growth, leaf and pollen development, and mitochondrial membrane potential maintenance [12,13,14]. Here, we showed that AtVDAC1 acts as a repressor during the floral transition, the *atvdac1-5* and *atvdac1* mutants have early-flowering phenotypes, and *AtVDAC1* is expressed ubiquitously in *Arabidopsis*. To identify the flowering pathway integrators that may be responsible for AtVDAC1 functions during the floral transition, an RNA-seq analysis was performed, and DEGs were identified between WT and *atvdac1-5* mutant seedlings during the floral transition. A GO term enrichment analysis of these DEGs showed that the top five dominant GO terms in cellular component were “mitochondrion”, “mitochondrial outer membrane”, “mitochondrial inner membrane”, “mitochondrial envelope”, and “vacuolar membrane” (Figure 3C), which indicated the important roles of AtVDAC1 in mitochondria.

FT protein movement may be the main reason for the early-flowering phenotype of *atvdac1-5*. First, transcriptomic analyses between WT and *atvdac1-5* mutant seedlings revealed that none of the DEGs were involved in floral transition. Second, multiple protein –protein interaction assays demonstrated that AtVDAC1 interacts with the FT protein in vivo.

In *Arabidopsis*, the FT protein is generated in the companion cells of vascular tissues. The translocation of FT into sieve elements and its movement to the shoot apical meristem through the phloem requires FT to interact with multiple proteins. When FT protein moves to the shoot apical meristem cells, FT interacts with 14-3-3 proteins in the cytoplasm, and then, the FT–14-3-3 complex enters the nucleus, where it interacts with FD to form florigen activation complexes. The complexes bind to the promoter regions of floral meristem identity genes (e.g., AP1), thereby activating their gene expression to promote flowering [26,27]. Because VDAC proteins play important roles in metabolite transport between mitochondria and the cytoplasm [2,3], we speculated that AtVDAC1 interacts with the FT protein in the cytoplasm and then translocates it into mitochondria. In the mitochondria, the FT protein cannot interact with the 14-3-3 protein or FD protein, thereby failing to activate the floral meristem identity genes. When the function of AtVDAC1 was interrupted, more FT protein interacted with the 14-3-3 protein and then entered the nucleus to interact with FD. More florigen activation complexes formed in the nucleus and then increased the floral meristem identity genes’ expression levels, resulting in the early-flowering phenotype of the *atvdac1-5* mutant. Although the expression levels of the floral meristem identity genes were not altered in the *vdac1-5* mutant at 9 DAG, the expression of *AP1* and *LFY* were all increased in *vdac1-5* and *atvdac1* mutants at 13 DAG (Figure 4A,B), possibly because at the early stage the expression of *AP1* and *LFY* were all at a low level.

Here, we speculate that AtVDAC1 prevented flowering by translocating the FT protein into mitochondria. However, *atvdac1-5 ft-10* double mutants flowered earlier than *ft-10* (Figure 5D); consequently, we believe that AtVDAC1 may also interact with other components to regulate flowering, such as TWIN SISTER OF FT (TSF), the closest homolog of FT. TSF and FT probably perform redundant roles, and AtVDAC1 may also translocate the TSF protein into mitochondria to prevent flowering. Further studies should focus on the other interacting partners of AtVDAC1 that are associated with floral-transition regulation.

## 4. Materials and Methods

### 4.1. Plant Materials and Growth Conditions

The Arabidopsis plants were grown under long-day (16-h/8-h, light/dark) conditions at 23 °C with a relative humidity of 75%. The plants were grown under cool-white fluorescence lamps, with a light intensity of 100 µmol·m^−2^·s^−1^ at the soil surface. Transgenic plants were generated through Agrobacterium tumefaciens-mediated transformation using the floral dipping method. Briefly, the gAtVDAC1-3FLAG and gAtVDAC1-GUS constructs were transformed independently into A. tumefaciens strain GV3101. Then, A. tumefaciens was cultured overnight in a 28 °C incubator at 200 rpm to reach OD600 = 1.8. Afterward, they were transformed independently into atvdac1-5 mutants using the floral dipping method. The atvdac1-5 mutants being used for transformation were approximately 4 weeks old with plenty of inflorescences. Developing floral tissues were dipped into an *Agrobacterium* solution containing 5% sucrose and Silwet-77 (500 μL L^−1^). After inoculation, the plants were placed under a plastic dome for 24 h and then grown under normal conditions. Seeds were obtained following self-pollination. Transformants containing gAtVDAC1-3FLAG and gAtVDAC1-GUS, independently, were selected on MS medium supplemented with hygromycin (30 mg L^−1^). Two independent homozygous T3 lines with hygromycin resistance were chosen for further studies.

### 4.2. Plasmid Construction

To construct *gAtVDAC1-3FLAG* and *gAtVDAC1-GUS*, a 4.5-kb *AtVDAC1* genomic fragment (*gAtVDAC1*) containing the 2.6-kb upstream sequence and 1.9-kb full-coding sequence plus introns was amplified and subsequently cloned into binary vector pCAMBIA1300, which already contained the 3FLAG and GUS tags, respectively. The coding sequences of *FT* and *AtVDAC1* were amplified and subsequently cloned into the binary vectors pCAMBIA1300-35S:nLUC and pCAMBIA1300-35S:cLUC, respectively, to create *35S:FT-nLUC* and *35S:cLUC-AtVDAC1*. The primers used for plasmid construction are listed in Table S2.

### 4.3. Yeast Two-Hybrid Assay

To construct the vectors for the yeast two-hybrid assays, the coding regions of AtVDAC1 and FT were amplified and cloned into pGADT7 and pGBKT7, respectively. The yeast two-hybrid assay was performed using the Yeastmaker Yeast Transformation System 2, in accordance with the manufacturer’s instructions (Clontech, Palo Alto, CA, USA). The yeast two-hybrid assay was performed three times. All the primers used for generating these constructs are listed in Table S2.

### 4.4. LCI Assays

*Agrobacterium tumefaciens* GV3101, carrying the indicated plasmids, was prepared in infiltration medium (MS, 10 mM MES, and 150 μM acetosyringone) at OD600 = 0.5. The cultures were transiently infiltrated into *N. benthamiana* leaves, and the LUC luminescence intensity was detected using a ROPER CA2048B (ROPER Scientific). The LCI assays were performed with three biological replicates.

### 4.5. GUS Staining

GUS staining was carried out using a published protocol [28] with minor modifications. Seedlings were incubated with GUS staining solution (100 mM sodium phosphate buffer, pH 7.0, 10 mM ethylenediaminetetraacetic acid disodium salt, pH 8.0, 1 mM potassium ferrocyanide, 1 mM potassium ferricyanide, 0.5% (*v*/*v*) Triton X-100, 20% (*v*/*v*) methanol, and 0.5 mg/mL X-Gluc) under vacuum conditions and then stained at 37 °C for 6 h. The stained tissues were cleared of chlorophyll using an ethanol series, placed in a clear solution (chloral hydrate/water/glycerol = 8 g/3 mL/1 mL), and observed under a light microscope.

### 4.6. RNA-seq Analyses

Total RNA was extracted from the aerial part of seedlings with an RNAprep Pure Plant Kit (TIANGEN, Beijing, China) and analyzed by a NanoDrop and an Agilent 2100 bioanalyzer (Thermo Fisher Scientific, MA, USA). Oligo (dT) magnetic beads were used to purify mRNA, and then, the mRNA was sheared into small pieces in a fragmentation buffer. The first-strand cDNA was synthesized using random hexamer-primed reverse transcription, and then, second-strand cDNA was synthesized. Afterward, adapters were added to the double-stranded cDNA, and the resulting cDNA fragments were amplified by PCR. The PCR products were purified and dissolved in EB solution. The PCR products from the previous step were heat-denatured and circularized to produce the final library. The final library was loaded and the reads were generated on the BGIseq500 platform (BGI-Shenzhen, China). The transcriptome datasets have been submitted to the NCBI (accession number PRJNA695037). The sequencing data were filtered and analyzed in accordance with a previous article [29]. The significance levels of genes and GO-term enrichment analyses were set at Q-value ≤ 0.05.

### 4.7. Expression Analysis

In total, 1 μg total RNA was reverse transcribed using a FastKing gDNA Dispelling RT SuperMix kit (TIANGEN, Beijing, China). Quantitative real-time PCR was performed on the CFX96 real-time PCR detection system (Bio-Rad) using the UltraSYBR Mixture (CWBio, Beijing, China). The expression of *TUBULIN 2* (*TUB2*) was used as an internal control. The expression analysis was performed with three biological replicates. All of the primers used for the expression analysis are listed in Table S2.

### 4.8. Flowering Time Measurement

Flowering time was calculated and represented as the average number of rosette leaves when the first flower appeared.

### 4.9. Arabidopsis Protoplast Isolation and Sub-Cellular Localization Assay

*Arabidopsis* protoplasts were isolated by following the previously published method [30]. SUC2:GFP-FT construct was transformed into *Arabidopsis* protoplast by the PEG4000-mediated method as described previously [31]. GFP, MitoTracker Red CMXRos (Eugene, OR, USA) and Mt-rk CD3-991 [32] were measured with a confocal laser scanning microscope ZEISS LSM 880 (Carl Zeiss, Oberkochen, Germany).

### 4.10. BiFC Analysis

The cDNAs of the proteins tested were cloned into pDOE-03 vector containing N- and C-terminal yellow fluorescence protein mVenus fragments for BiFC analysis as previously published [33].

### 4.11. Statistical Analysis

The experimental data were analyzed using two-tailed paired Student’s *t* tests with SPSS 12.0 software.

## Data Availability

The transcriptome datasets have been submitted to the NCBI (accession number PRJNA695037).

## Supplementary Materials

The following are available online at https://www.mdpi.com/article/10.3390/ijms222111603/s1.
