# Supplementary material for: VDAC1 Negatively Regulates Floral Transition in Arabidopsis thaliana
Source: Int J Mol Sci. 2021 Oct 27;22(21):11603. doi: 10.3390/ijms222111603 (PMC8584032; doi:10.3390/ijms222111603)
Supplement: Supplementary file 1 [file ijms-22-11603-s001.zip › Table S2 List of primers used in this study.pdf]

Table S2. List of primers used in this study

Primers for plasmid construction

**Construct name**	**Sequence (5'-3')**
gVDAC1-3FLAG	CAGCTATGACCATGATTACGGAATTCTTTGATGCCATATATATATG
	CTAGAGGATCCCCGGGTACCGAGCTCAGGCTTGAGTGCGAGAGCCA
gVDAC1-GUS	CGACGGCCAGTGCCAAGCTTTTTGATGCCATATATATATG
	CTCAGATCTACCATGGTACCAGGCTTGAGTGCGAGAGCCA
35S:FT-nLUC	ACGGGGGACGAGCTCGGTACCATGTCTATAAATATA
	CGCGTACGAGATCTGGTCGACAAGTCTTCTTCCTCC
35S:cLUC-VDAC1	GTACGCGTCCCGGGGCGGTACCATGGTGAAAGGTCC
	ACGAAAGCTCTGCAGGTCGACTCAAGGCTTGAGTGC
pGADT7-VDAC1	AAAAAACATATGATGGTGAAAGGTCCCGGTC
	AAAAAACTCGAGTCAAGGCTTGAGTGCGAGA
pGBKT7-FT	AAAAAACATATGTCTATAAATATAAGAGACCCT
	AAAAAAGTCGACCTAAAGTCTTCTTCCTCCGCAGCC
pDOE-03-NmVen210-FT	GTGGAGGTGGGTCAGGATCCATGTCTATAAATATAAGAGA
	AGGGCGCGCCCCATGGATCCCTAAAGTCTTCTTCCTCCGC
pDOE-03-VDAC1-CVen 210	GTAGTCACGTGACGTCCGGAATGGTGAAAGGTCCCGGTCT
	GCTCCACCAGAACCTCCGGAAGGCTTGAGTGCGAGAGCCA

Primers for quantitative real-time PCR

**Gene name**	**Sequence (5'-3')**
VDAC1	CGCTACCGTTGATGAGG
	TTGACAGTTGGGTTCTGAGTC
AP1	GAAGGCCATACAGGAGCAAA
	ACTGCTCCTGTTGAGCCCTA
LFY	ACGCCGTCATTTGCTACTCT
	CTTTCTCCGTCTCTGCTGCT
TUB2	ATCCGTGAAGAGTACCCAGAT
	AAGAACCATGCACTCATCAGC

Primers for semi-quantitative real-time PCR

**Gene name**	**Sequence (5'-3')**
VDAC1 F	CGCTACCGTTGATGAGG
VDAC1 R1	CCAGCATTGATCTTGGT
VDAC1 R2	GCCCACTCAAACCATGA

Primers for genotyping assays

Gene name	Sequence (5’-3’)
atvdac1-5	GGGAAAGATCAGTAGTTGCCC
	TCGTTGCTCATAATCTGGCTC
atvdac1	TTATTACAGGCCAACAATGCC
	GTGATTGGCTCCAATGTCTTG
ft-10	GGTGGAGAAGACCTCAGGAAC
	TTTTGGGAGACAAATTGATGC
SALK-LBb1.3	ATTTTGCCGATTTCGGAAC
08409-GABI	ATATTGACCATCATACTCATTGC
