# Supplementary figures and images for: VDAC1 Negatively Regulates Floral Transition in Arabidopsis thaliana
Source: Int J Mol Sci. 2021 Oct 27;22(21):11603. doi: 10.3390/ijms222111603 (PMC8584032; doi:10.3390/ijms222111603)

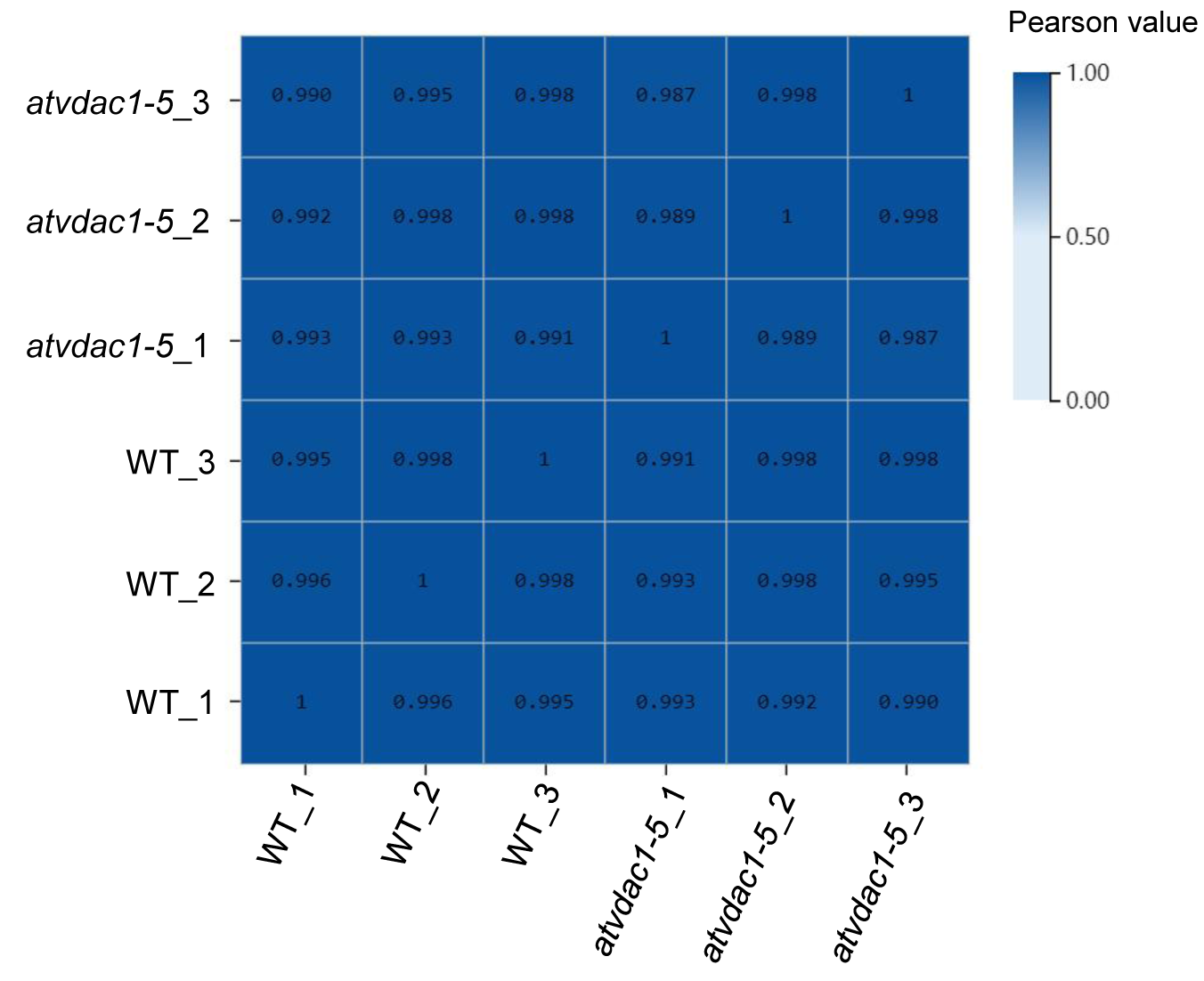

Supplement: Supplementary file 1 [file ijms-22-11603-s001.zip › Figure S1.tif]
